# Safety assessment of fluorescently labeled anti-EGFR Nanobodies in healthy dogs

**DOI:** 10.3389/fphar.2023.1266288

**Published:** 2023-09-14

**Authors:** Nayra Cristina Herreira do Valle, Simone Janssen, Marcus C. M. Stroet, Sofie Pollenus, Sonja Van den Block, Nick Devoogdt, Jens M. Debacker, Sophie Hernot, Hilde De Rooster

**Affiliations:** ^1^ Small Animal Department, Faculty of Veterinary Medicine, Ghent University, Ghent, Belgium; ^2^ Molecular Imaging and Therapy Research Group, Faculty of Medicine and Pharmacy, Vrije Universiteit Brussel, Brussels, Belgium; ^3^ Cancer Research Institute Ghent (CRIG), Ghent University, Ghent, Belgium; ^4^ Department of Nuclear Medicine, UZ Brussel, Brussels, Belgium; ^5^ Department of Head and Skin, Research Group Head and Neck Surgery, Ghent University, Ghent, Belgium

**Keywords:** EGFR, nanobody, single-domain antibody, toxicity, dogs, image-guided surgery, NIR fluorescence

## Abstract

**Introduction:** Surgical resection is one of the main treatment options for several types of cancer, the desired outcome being complete removal of the primary tumor and its local metastases. Any malignant tissue that remains after surgery may lead to relapsing disease, negatively impacting the patient’s quality of life and overall survival. Fluorescence imaging in surgical oncology aims to facilitate full resection of solid tumors through the visualization of malignant tissue during surgery, following the administration of a fluorescent contrast agent. An important class of targeting molecules are Nanobodies^®^ (Nbs), small antigen-binding fragments derived from camelid heavy chain only antibodies. When coupled with a fluorophore, Nbs can bind to a specific receptor and demarcate tumor margins through a fluorescence camera, improving the accuracy of surgical intervention. A widely investigated target for fluorescence-guided surgery is the epidermal growth factor receptor (EGFR), which is overexpressed in several types of tumors. Promising results with the fluorescently labeled anti-EGFR Nb 7D12-s775z in murine models motivated a project employing the compound in a pioneering study in dogs with spontaneous cancer.

**Methods:** To determine the safety profile of the study drug, three healthy purpose-bred dogs received an intravenous injection of the tracer at 5.83, 11.66, and 19.47 mg/m^2^, separated by a 14-day wash-out period. Physical examination and fluorescence imaging were performed at established time points, and the animals were closely monitored between doses. Blood and urine values were analyzed pre- and 24 h post administration.

**Results:** No adverse effects were observed, and blood and urine values stayed within the reference range. Images of the oral mucosa, acquired with a fluorescence imaging device (Fluobeam^®^), suggest rapid clearance, which was in accordance with previous *in vivo* studies.

**Discussion:** These are the first results to indicate that 7D12-s775z is well tolerated in dogs and paves the way to conduct clinical trials in canine patients with EGFR-overexpressing spontaneous tumors.

## 1 Introduction

In surgical oncology, fluorescence imaging is an important technique to enable perioperative visualization of tumoral tissue by differentiating it from surrounding healthy tissue (Mieog et al., 2022). For this purpose, several targeted molecules such as monoclonal antibodies (mAbs), antibody fragments, peptides and small molecules coupled with a fluorophore have been investigated in clinical and preclinical studies with promising results (Hernot et al., 2019). Targeted contrast agents present increased specificity and imaging contrast over non-targeted fluorophores such as indocyanine green (Lauwerends et al., 2021), improving discrimination between malignant and non-malignant tissue. Ergo, a folate analogue paired with a NIR fluorescent dye, pafolacianine, was recently approved for ovarian and lung cancer surgery under Fast Track designation by the FDA (Tanyi et al., 2022).

One of the most explored targets in the field of fluorescence-guided surgery is the epidermal growth factor receptor (EGFR), as it is overexpressed in several types of cancer, including breast, lung, bladder, brain, and head and neck cancer (Talukdar et al., 2020). Understandably, mAbs are often proposed as molecular targeting agents given their natural ability to recognize specific epitopes (Panowski et al., 2014; Cai et al., 2020). Clinically approved EGFR-targeting mAbs, namely, cetuximab and panitumumab, were repurposed as fluorescent contrast agents after conjugation with the near-infrared (NIR) fluorophore IRDye800CW^®^ and investigated in various human studies (Rosenthal et al., 2015; Marston et al., 2019; Nishio et al., 2020; Voskuil et al., 2020). These early phase clinical trials have demonstrated their capacity to demarcate both *in situ* and *ex vivo* tumor lesions in head and neck, pancreatic, lung, and brain cancer patients. Despite these compelling results, mAbs suffer from slow kinetics and non-specific uptake due to their relatively large size, posing a limitation in their application as imaging agents (Rosenthal et al., 2017).

In an effort to address these shortcomings, several alternative low-molecular weight EGFR-targeting molecules are currently being investigated due to their pharmacokinetic properties. For instance, ABY-029, an IRDye800CW labeled anti-EGFR Affibody^®^, was validated preclinically (Samkoe et al., 2017; 2019) and is currently being employed in a Phase 0 clinical trial in head and neck cancer patients (NCT03282461) (Chen et al., 2023). Another promising platform molecule is the Nanobody^®^ (Nb), a molecule of 10–14 kD in size, derived from the antigen-binding site of camelid heavy chain only antibodies. The first clinically available Nb, caplacizumab, was approved in 2018 for the treatment of acquired thrombotic thrombocytopenic purpura, and many more are under clinical investigation for distinct therapeutic and diagnostic applications (Debie et al., 2019; Jovčevska and Muyldermans, 2020; Awad et al., 2022).

In the context of fluorescence-guided surgery, the anti-EGFR Nb 7D12 coupled with IRDye800CW ^®^ has been previously investigated in mice biodistribution and imaging studies, showing successful tumor accumulation in xenografts and orthotopic models (Oliveira et al., 2012; Van Driel et al., 2014). However, IRDye800CW presents significant limitations for tracer development due to high non-specific background signals (Debie et al., 2017; Li et al., 2020). The novel cyanine-based dye s775z has been optimized to limit self-aggregation and non-selective binding by sterically shielding the heptamethine backbone of the fluorochrome with two triethylene glycol arms. Previous studies in mice reported its superior photostability and contrast when coupled with cancer-targeting moieties compared to commonly used NIR fluorophores (Li et al., 2020; Gamage et al., 2021; Schreiber et al., 2021).

Following promising studies performed by our group employing 7D12-s775z in murine cancer models (Declerck et al., 2023), we aim to confirm the applicability of this compound in canine cancer patients. As a complementary model between rodents and humans in translational studies, dogs with spontaneous cancer could be an asset in increasing clinical trials’ success rate. Dogs and humans share numerous similarities in tumor behavior, exposure to environmental risk factors, and molecular landscape (Paoloni and Khanna, 2008; LeBlanc and Mazcko, 2020). Unlike mice, the body size of dogs also allows for comparative surgical studies and successful trials in dogs could benefit both human and canine cancer patients. To date, no study has described the tolerability of intravenously injected Nbs in dogs. Thus, we aimed to determine the safety profile of 7D12-s775z in this species, in preparation for a proof-of-concept study in dogs with spontaneous cancer.

## 2 Materials and methods

### 2.1 Production of 7D12 and preparation of 7D12-s775z

The anti-EGFR Nb 7D12 (Roovers et al., 2011), genetically engineered with a carboxyterminal hexahistidine tag, was produced by the VIB Protein Core facility (Ghent, Belgium) in *Pichia pastoris* according to a standard 6L-flask culture protocol and purified via immobilized metal affinity chromatography and size-exclusion chromatography. The Nb was stored at −20 °C in 1 mL aliquots at a concentration of 4.68 mg/mL in PBS until further use. 7D12 was subsequently conjugated with the NIR dye s775z via amine-reactive NHS chemistry. The conjugation reaction and purification process were performed using decontaminated equipment and sterilized vials; all handlings were carefully documented. High purity materials were purchased at Sigma Aldrich (St. Louis, MO, United States) unless stated otherwise. All solutions were freshly prepared from clinical-grade water for injection (Baxter, Glenview, IL, United States) in Nalgene Biotainers (Thermo Fisher Scientific, Waltham, MA, United States), and were filtered through a 0.22 µm PES filter before use. Sterile PBS was prepared by dissolving tablets (Millipore Sigma, Burlington, MA, United States).

For every production cycle, 32.8 mg of 7D12 Nb (7 mL) was concentrated using a centrifugal concentrator (Vivaspin 5000 HY, Sartorius, Göttingen, Germany) to a volume of 1.6–1.7 mL. The solution was buffered to pH 8.5 by the addition of 500 µL of 1 M K_2_HPO_4_ (pH 8.5). A 4-fold molecular excess of s775z-NHS (s775z-NHS, Fluoroprobes, Scottsdale, AZ, United States) dissolved in DMSO at 100 mg/mL was added in 10 µL portions. The resulting mixture was incubated for 2 h at room temperature, protected from light exposure. The fluorescently labeled Nb was then purified by size-exclusion chromatography (SEC) on an NGC chromatography system (Bio-Rad Laboratories, Hercules, CA, United States) equipped with a HiLoad 16/600 Superdex 75 pg column (Cytiva, Marlborough, MA, United States) with PBS as elution buffer (1.0 mL/min). Detection at 280 and 775 nm was used for the analysis of the Nb and dye absorbance, respectively. The concentration of the bioconjugate and the degree of fluorophore conjugation in each collected fraction were determined by measuring the absorbance of the dye at 775 nm (extinction coefficient 231,000 M^-1^cm^-1^) and the Nb at 280 nm (extinction coefficient 34,045 M^-1^cm^-1^) with a NanoDrop 2000 (Thermo Fisher Scientific). A correction factor for the absorption by the dye at 280 nm was applied, by subtracting 3% of the absorption at 775 nm from the absorption recorded at 280 nm. Finally, relevant fractions containing 7D12-s775z with an average degree of labeling (DOL) of 1 were collected and pooled in sterile, light-protected vials. The samples were aliquoted and stored at −20 °C until further use.

### 2.2 Quality control of 7D12 and 7D12-s775z

Quality control was performed on both unlabeled and labeled Nb by analytical SEC (50–100 µg Nb in 250 µL PBS) using a Superdex 75 Increase 10/300 GL column (GE Healthcare, Chicago, IL, United States) with PBS as elution buffer (0.8 mL/min). The purity of the (un)labeled Nb was further investigated by SDS-PAGE. Hereto, 0.5, 2, and 10 µg were loaded on a 16% Tris-Glycine Mini Gel (WedgeWell format, 10-well, Novex, Thermo Fisher Scientific) under reducing (1:1 dillution 2x Laemmli buffer, Bio-Rad, Hercules, CA, United States) and non-reducing conditions. 0.1 µg Bovine Serum Albumine (BSA) was loaded as detection limit reference. Prior to visualization of the proteins with Coomassie blue (Abcam, Cambridge, UK), gels were scanned under the Odyssey fluorescence scanner (LI-COR Biosciences, Lincoln, NE, United States) using the 800 nm channel. Western blot was performed by blotting the gel onto a Whatman paper (Bio-Rad Laboratories). The paper was incubated for 1 h with HRP-conjugated anti-His Ab (1:10,000 dilution, 130–092–783, Miltenyi Biotec, Bergisch Gladbach, Germany) and subsequently washed and incubated in freshly prepared ECL solution (Cytiva) for 5 minutes, after which chemiluminescent blotting images were recorded with an Amersham Imager 680RGB (General Electric, Boston, MA, United States).

Mass spectrometry (MS) was performed by loading 1 µg of (un)labeled Nb on a Poroshell 300SB-C8 column (1 × 75 mm, 5 μm particles, Agilent, Santa Clara, CA, United States), using a linear 5 min gradient from 95% solvent A (0.1% formic acid, 0.05% trifluoroacetic acid in water) to 90% solvent B (0.1% formic acid, 0.05% trifluoroacetic acid in acetonitrile). The mass spectrometer was operated in MS mode at a resolution of 60,000 (at m/z 400) in a mass range from 600 to 4,000 m/z. The recorded spectra were deconvoluted with the Xtract™ algorithm in the Freestyle software (Thermo Fisher Scientific). The spectral characteristics of 7D12-s775z (1 μM, PBS) were recorded in a quartz cuvette with a spectrofluorophotometer (RF-6000, Shimadzu, Kyoto, Japan). The excitation spectrum from 200 to 900 nm at 1 nm steps was recorded at an emission wavelength of 794 nm. The wavelength with the highest excitation signal was used to record the corresponding emission spectrum. The spectra were normalized to the highest recorded excitation or emission signal.

Endotoxin levels were determined by a Limulus Amebocyte Lysate test (LAL, Endosafe-PTS SN 2831, Charles River, Wilmington, MA, United States); the endotoxin level was normalized to the concentration of endotoxin units per mg of Nb (EU/mg), and 5 EU/mg was set as the maximally accepted endotoxin level for further use.

After 6 months of storage at −20°C in the dark, a stability assessment of 7D12-s775z was performed by SEC, MS, SDS-PAGE, and by recording the spectral properties. Additionally, the serum stability of the compound was assessed *in vitro.* 100 μg of 7D12-s775z was incubated in a final volume of 150 µL of canine serum up to 24 h at 37 °C and analyzed via SEC on a Superdex 75 5/150 GL column (GE Healthcare) with PBS as running buffer eluting at 0.3 mL/min.

### 2.3 Surface plasmon resonance

The binding affinity of 7D12 and 7D12-s775z towards EGFR, as well as the association (k_a_) and dissociation rates (k_d_), were determined using surface plasmon resonance (SPR, Biacore T200, GE Healthcare). A concentration of 15 μg/mL of canine or 10 μg/mL of human EGFR-His recombinant protein (Sino Biological, Beijing, China) was first immobilized on a series S Sensor Chip CM5 (Cytiva). (Un)labeled Nbs were then injected consecutively in two-fold serial dilutions ranging from 250 nM to 2 nM. The association step was allowed for 100 s, the dissociation step for 600 s. Regeneration was performed during 20 s at 30 μL/min using 0.1 M glycine at pH 2.5 followed by a stabilization period of 180 s. The kinetic rate constants were determined by mathematical fitting using the 1:1 binding with drift and RI2 model proposed by the Biacore Evaluation Software (Cytiva), and the k_d_/k_a_ ratio was used to determine the equilibrium dissociation constant (K_D_).

### 2.4 Cell culture and flow cytometry

The affinity of 7D12-s775z towards EGFR was further assessed by flow cytometry. The canine mammary carcinoma cell line P114, kindly provided by Prof. van Nimwegen (Department of Clinical Sciences, Utrecht University, Netherlands), was cultured in 1:1 DMEM:F12 medium (Sigma-Aldrich, St. Louis, MO, United States) supplemented with 10% Fetal Bovine Serum (FBS, Pan Biotech, Aidenbach, Germany) and 1% PenStrep (Gibco Life Technologies, Waltham, MA, United States). Human FADU cells (ATCC, Manassas, VA, United States) were cultured in MEM (Gibco Life Technologies) supplemented with 10% FBS and 1% PenStrep. P114 cells were pre-treated with canine Fc-block (14–9162–42, Thermo Fisher Scientific) before further use. P114 and FADU cells (100,000 per tube) were washed with FACS-buffer (PBS with 1% BSA and 0.02% sodium azide) and incubated with 7D12-s775z, a control non-targeting Nb, R3B23-s775z (Lemaire et al., 2014), cetuximab-Alexa Fluor 647 (in-house prepared by labeling cetuximab with Alexa Fluor 647 via lysine reactive NHS-chemistry), or isotype control IgG-Alexa Fluor 647 (DDXCH01A647-100, Novux Biologics, Abingdon, UK) at a concentration of 800 nM for 1 h at 4°C, shielded from light exposure. Binding of AF467-labeled cetuximab and IgG isotype control Ab was detected directly. For detection of Nb binding, cells were washed again and resuspended in 100 µL of anti-His-PE Ab (1:50, GG11-8F3.5.2, Miltenyi Biotec) for 10 min as secondary staining. Finally, all cells were washed, resuspended, and analyzed using the FACS Celesta flow cytometer (BD Biosciences, Franklin Lakes, NJ, United States). The FACS data were analyzed using FlowJo software package (BD Biosciences, San Jose, CA, United States), and nonviable cells were excluded using forward versus side scatter analysis.

### 2.5 Animals and ethics

The animal study was reviewed and approved by the Ethical Committee of the Faculties of Veterinary Medicine and Bioengineering Science (Institutional Animal Care and Use Committee; EC 2021–084) and was in accordance with the Belgian Royal Decree of 29 May 2013 and EU directive 2010/63/EU. Three healthy 6-year-old purpose-bred Beagle dogs (Ludwig-Maximilians-Universität München, Germany), two females and one male, with a body weight of 12, 13.4 and 15.9 kg, respectively, were enrolled in this study. Health was screened before each Nb administration through physical examination by a veterinarian and blood and urine analysis were performed under standard operating veterinary procedures. Physical examination consisted of the assessment of heart rate, respiratory rate, systolic blood pressure, rectal temperature, mucosal color, hydration status, and capillary refill time. Blood was collected from the jugular vein and analyses included complete blood count, serum biochemistry (albumin, alkaline phosphatase, alanine transaminase, urea, creatinine, globulin, glucose, total protein), and coagulation tests (prothrombin time and activated partial thromboplastin time). Urine was collected through cystocentesis, and density, pH, white and red blood cells, protein, glucose, ketone, urobilinogen, and bilirubin levels were measured.

### 2.6 Experimental design

All dogs received three escalating intravenous doses of 7D12-s775z, 5.83, 11.66, and 19.47 mg/m^2^, each separated by a 14-day wash-out period. The animals had no access to food for at least 12 h before each administration and were sedated immediately before the procedure using butorphanol (0.3 mg/kg) intravenously to ensure animal welfare and to emulate preoperative conditions. Water was offered *ad libitum*. Physical examinations were performed before and at 2′, 5′, 10′, 30′, 1, 2, 4, 8, 12, 18 and 24 h following injection, where the heart and respiratory rate, systolic blood pressure, and rectal temperature were monitored. During the 14-day wash-out periods, the animals were closely observed for adverse clinical symptoms. Blood and urine were collected 24 h after intravenous Nb administration and the same parameters described in 2.5. were analyzed.

### 2.7 Fluorescence imaging

Fluorescent images were acquired from the oral mucosa before and after injection at predefined time points (0.5, 1, 2, 4, 8, and 24 h) using the Fluobeam^®^ 800 imaging system (FLUOPTICS^©^, Grenoble, France) in a dark room at a fixed distance of 20 cm. The exposure time was set to 60 ms and only raw data were collected. Mean fluorescence intensity (MFI) was determined using the image processing software Fiji (Schindelin et al., 2012) after manually selecting the region of interest (ROI). ROI was selected at the labial vestibule above the mucogingival junction, corresponding to the alveolar mucosa, a highly perfused and naturally EGFR-expressing tissue.

## 3 Results

### 3.1 Production of 7D12 and preparation of 7D12-s775z

**FIGURE 1:**
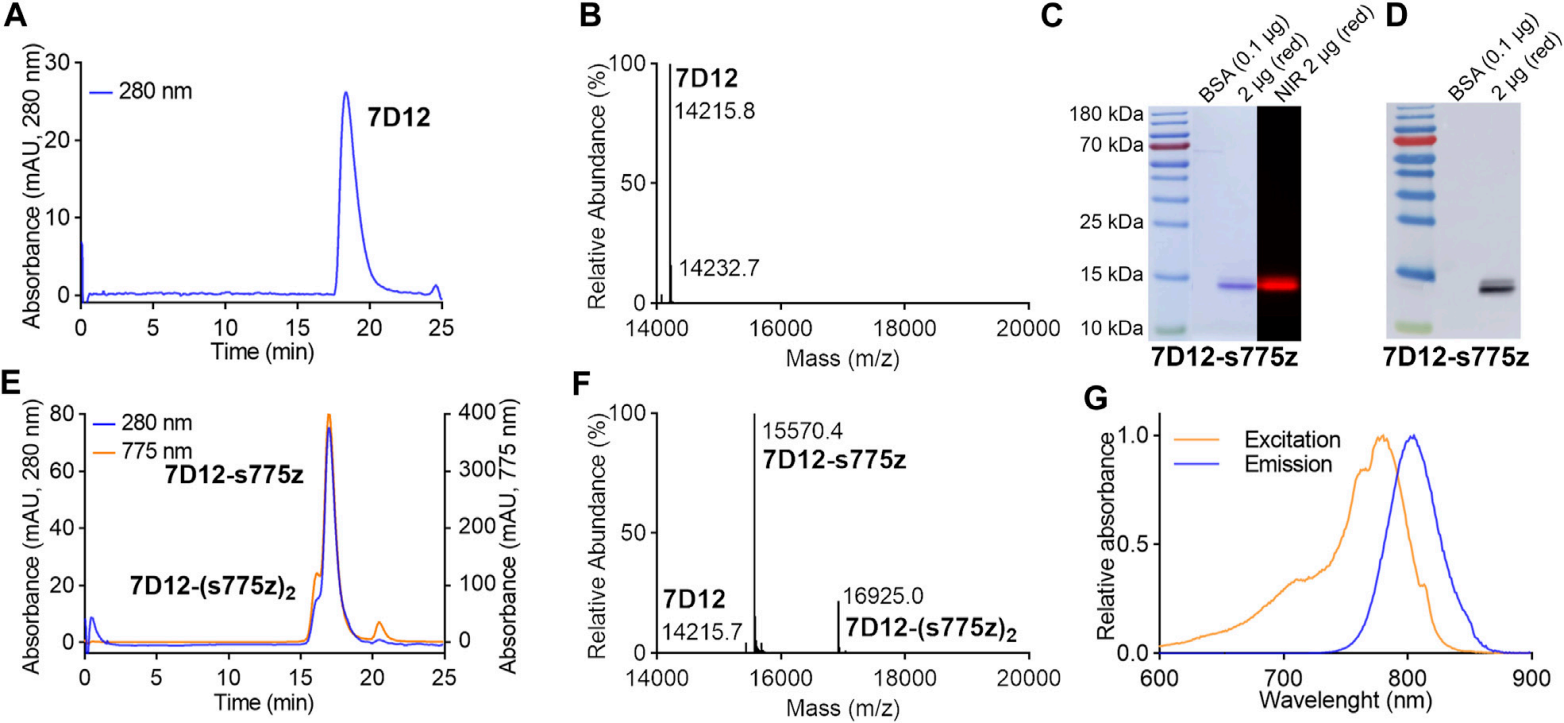
Chemical characterization of (un)labeled Nb 7D12. **(A)** Analytical SEC chromatogram of 7D12 (50 µg). The readout is in absorption at 280 nm (blue line). **(B)** Mass spectrum of 7D12, mass calculated: 14,231.75. m/z found was 14,215.76 [M-NH_3_+H]^+^, 14,232.77 [M + H]^+^, **(C)** Coomassie and NIR-fluorescence detection on SDS-PAGE of 7D12 at 2 µg under reducing conditions (red). 0.1 µg of bovine serum albumine (BSA) is included as minimum detection limit. **(D)** Chemiluminescence Western blot detection of His-tag on the Nb with Anti-His-tag antibody-HRP. **(E)** Chromatogram of analytical SEC of 7D12-s775z (100 µg). The readout is in absorption at 280 nm (blue line) and at 775 nm (orange line). **(F)** Mass spectrum of 7D12-s775z, mass calculated: 15,587.4, mass found 15,570.4 [M-NH_3_+H]^+^. **(G)** Spectral excitation spectrum (yellow line) and emission spectrum (blue line).

The 6 L *Pichia Pastoris* flask culture process yielded 360 mg of the 7D12 Nb. Purity of the compound was assessed by SEC and SDS-PAGE (Figures 1A,C and Supplementary Figures S1A,B), on which only a single peak/band was detected at the expected retention time/migration distance. The Nb’s molecular weight was confirmed by MS (m/z 14,215.0, Figure 1B) and its identity was established by Western blot through the detection of the His-tag at the carboxy-terminal end of the Nb (Figure 1D and Supplementary Figures S1C,D). The endotoxin concentration of the starting product evaluated by the LAL method corresponded to less than 0.22 EU/mg.

Next, the Nb was conjugated with the fluorescent dye s775z in six batches of ±30 mg each. Quality control of each batch was performed before release (Supplementary Table S1). Conjugation on the primary amines of the Nb yielded a mixture of Nbs with different DOL, up to four dyes per Nb. After SEC purification (Supplementary Figure S2) and collection of only the fractions corresponding mainly to Nbs conjugated with one dye, a DOL of 1.0 ± 0.1 was obtained as calculated by spectroscopy. This was confirmed by MS showing a prominent peak at m/z 15,570.4 (Figure 1F) and only minor peaks for unlabeled Nbs (m/z 14,215.7) or Nbs conjugated with two dyes (m/z 16,925.0). Furthermore, the bands at approximately 14 kDa on SDS-PAGE, corresponding to the conjugated Nbs, were also detectable with fluorescence imaging (Figure 1C). The maximal excitation and emission wavelengths of 7D12-s775z in PBS were 780 nm and 803 nm, respectively (Figure 1G). The endotoxin levels never exceeded the maximally accepted threshold of 5 EU/mg. The overall recovery yield of 7D12-s775z was 59% ± 17% (Supplementary Table S1). The stability of 7D12-s775z was assessed after 6 months of storage at −20°C, and no signs of degradation were observed on SEC and SDS-PAGE. Moreover, the concentration and spectral properties remained unchanged compared to what was measured at the time of preparation (Supplementary Figure S3). 7D12-s775z shows no serum protein binding and remains stable for at least 1 h in serum at 37 °C. After 3 h, some degradation is observed (Supplementary Figure S6).

### 3.2 Functionality assessment of 7D12 and 7D12-s775z

**FIGURE 2:**
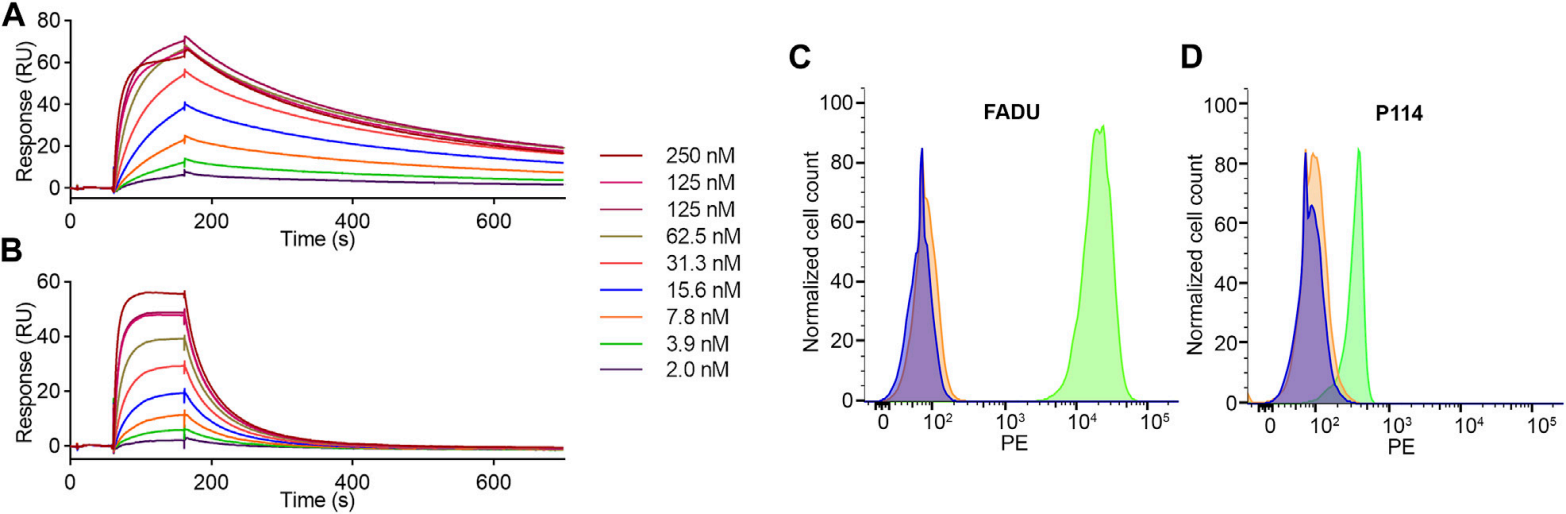
Assessment of binding kinetics of 7D12-s775z to human **(A)** and canine **(B)** recombinant EGFR via SPR analysis. Flow cytometry analysis on human FADU cells **(C)** and canine P114 cells **(D)**. Non-stained cells (purple), binding of control Nb (orange) and 7D12-s775z (green).

Affinity and binding kinetics of 7D12-s775z to both canine and human EGFR were determined by SPR and compared to the values for the unlabeled Nb. Resulting sensorgrams are shown in Figure 2; the K_D_, k_on_ and k_off_-values are presented in Table 1. The 7D12 Nb is confirmed to be cross-reactive, with a slightly lower affinity for canine recombinant EGFR (29.3 nM) than for human EGFR (5.41 nM). This is attributed to a 7-8-fold faster dissociation rate, while the association rate remains similar. After labeling with s775z, the affinity of the Nb is only minimally impacted (31.6 nM ± 3.9 and 11.1 ± 2.5 nM for canine and human EGFR, respectively) as compared to the unlabeled Nb (Table 1, and Supplementary Figure S4).

**TABLE 1:** Binding kinetics and affinity of 7D12 and 7D12-s775z to human and canine recombinant EGFR via SPR analysis.

	Human recombinant EGFR			Canine recombinant EGFR		
	k_on_ (10^6^ M^-1^s^-1^)	k_off_ (s^-1^)	K_D_ (nM)	k_on_ (10^6^M^-1^s^-1^)	k_off_ (s^-1^)	K_D_ (nM)
7D12	0.674	0.004	5.4	1.091	0.032	29.3
7D12-s775z	0.410 ± 0.072	0.004 ± 0.001	11.1 ± 2.5	0.907 ± 0.110	0.028 ± 0.001	31.6 ± 3.9

The cross-reactivity of 7D12 was further validated using flow cytometry on two cancer cells expressing either human or canine EGFR. Indeed, an increase in mean fluorescence intensity (ΔMFI) was seen for both FADU and P114 cells after incubation with 7D12-s775z as compared to unstained cells or cells incubated with a non-targeting control Nb (Figures 2C,D). EGFR expression on these cells was confirmed using Alexa Fluor 647-labeled cetuximab as a positive control (Supplementary Figures S5A,B).

### 3.3 Safety assessment

**FIGURE 3:**
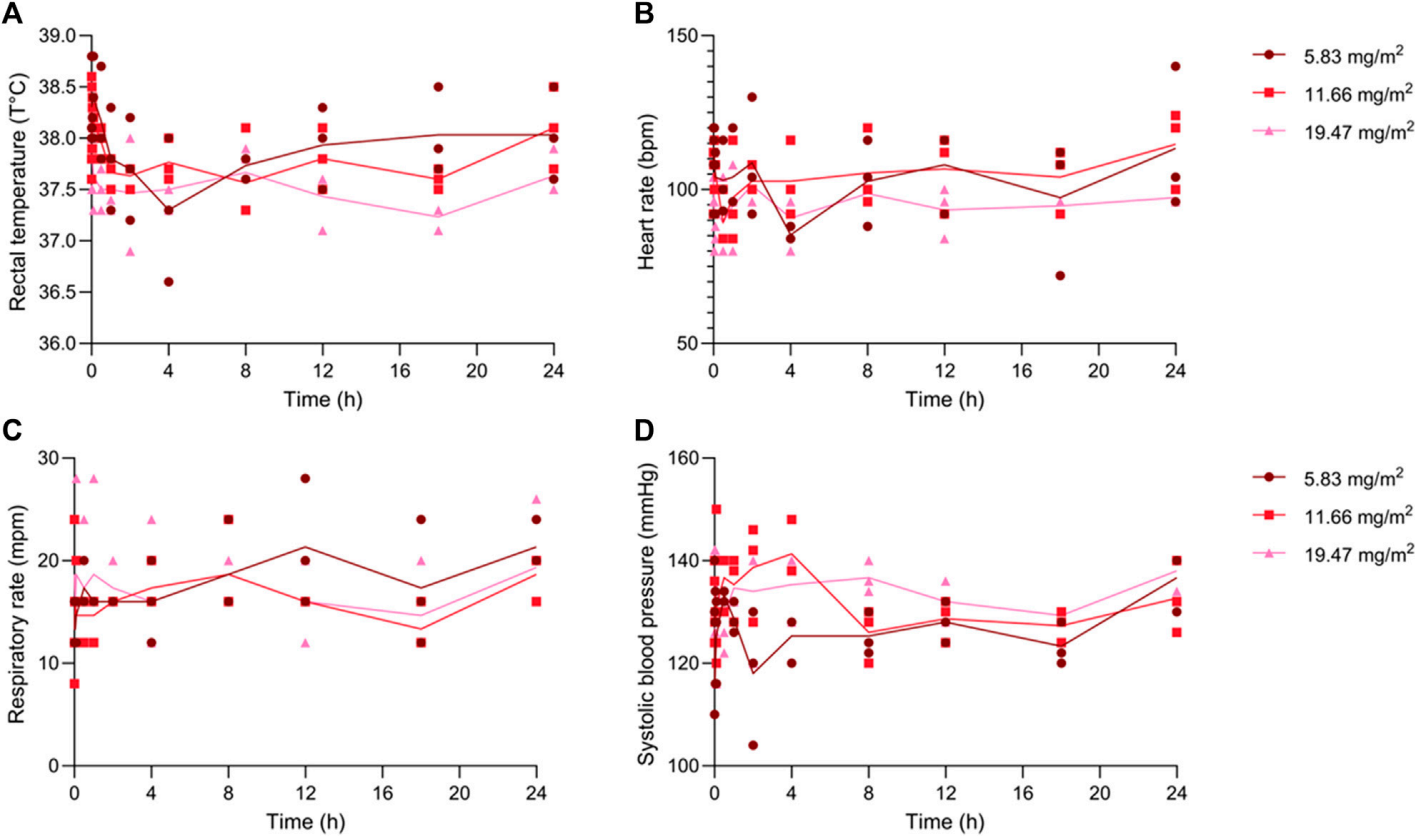
Mean **(A)** rectal temperature, **(B)** heart rate, **(C)** respiratory rate and **(D)** systolic blood pressure of all three dogs after each dose.

Before and after each intravenous injection of the study drug, thorough physical examinations were performed up to 24 h post-administration. Vital parameters remained within the normal range (Figures 3A–D). Furthermore, during the 14-day wash-out periods, the animals were closely monitored, and no adverse clinical effects were observed. Blood and urine were collected before and 24 h after injection and did not present significant alterations on the analysis panels. All data for the individual dogs are presented in the (Supplementary Table S2-S7).

### 3.4 Fluorescence imaging

**FIGURE 4:**
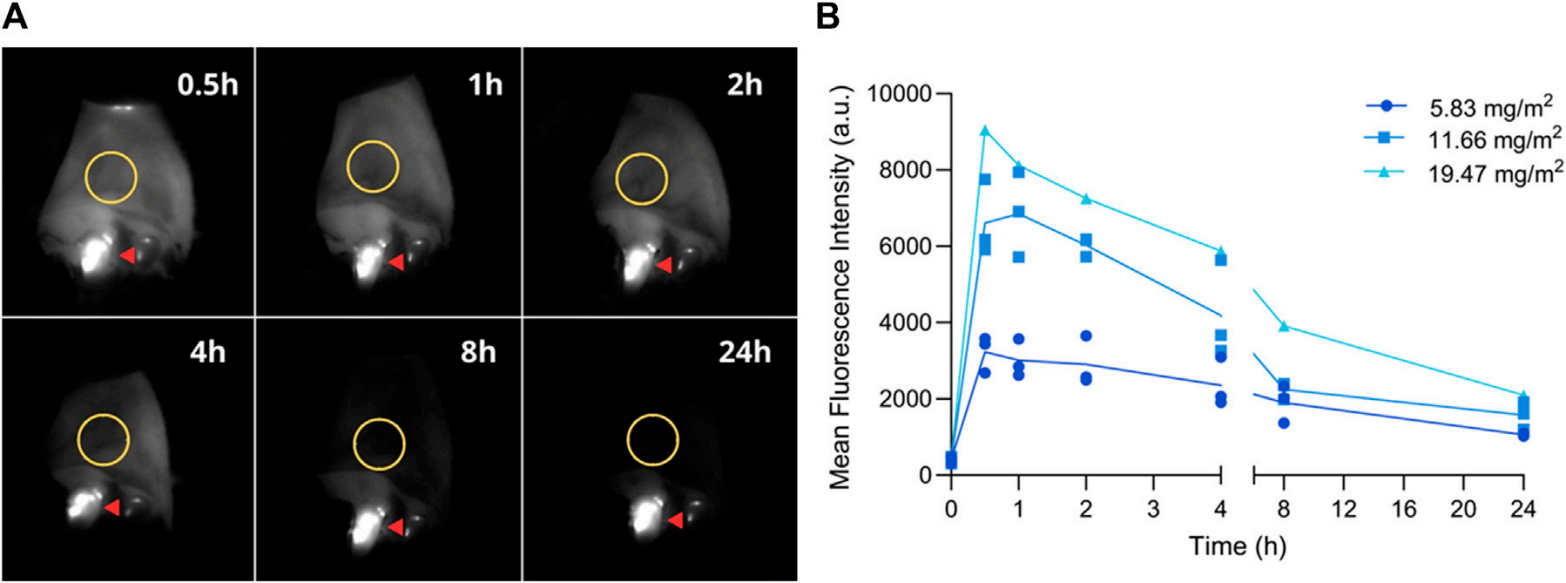
Fluorescence imaging of the oral mucosa at pre-established timepoints. **(A)** Representative fluorescent images; ROI is delineated with a yellow circle. Fluorescence observed on teeth originates from dental plaque (red arrow). **(B)** Mean fluorescence intensity before (0) and 0.5, 1, 2, 4, 8, and 24 h post-injection. Graph depicts the MFI on the oral mucosa of all three dogs after each dose.

Fluorescent images were acquired from the oral mucosa before and after injection at predefined time points (0.5, 1, 2, 4, 8, and 24 h). Images were processed and a ROI was selected to determine MFI (Figure 4A). Fluorescence intensity reached its maximum between the 1- and 2-h time point, decreasing subsequently until 24 h after injection (Figure 4B and Supplementary Figure S5).

## 4 Discussion and conclusion

Clear surgical margins remain one of the main outcome measures in cancer patients undergoing tumor resection. To complement the surgeon’s ability to visualize and remove tumoral tissue, molecular imaging could be of important added value (Stewart and Birch, 2021). Among targeted contrast agents that might be employed for fluorescence-guided surgery, the fluorescently labeled anti-EGFR Nb 7D12-s775z emerges as a promising tracer according to murine studies (Declerck et al., 2023). Whereas clinical translation of a new compound is costly and time-consuming, experiments in dogs can be a valuable intermediate step to evaluate the efficacy of a specific drug. Despite the fact that murine models are valuable assets in preclinical research, the number of novel drugs undergoing clinical translation is very restricted (DiMasi et al., 2010). Limitations such as low genetic diversity and homogeneity of induced tumors are the probable culprit for the low success rate in translational studies (Cheon and Orsulic, 2011). To close the gap between mice and humans, dogs with spontaneous cancer arise as an important complementary step. Due to similarities, including molecular landscape, tumor behavior, and even exposure to environmental risk factors, new techniques tested in naturally tumor-bearing dogs have the potential of benefiting both human and canine cancer patients (Paoloni and Khanna, 2008; LeBlanc and Mazcko, 2020). However, in order to administer a novel tracer to canine patients, the safety of the compound must be determined in healthy purpose-bred dogs. The current study is the first to report intravenous administration of Nbs in dogs and to assess the tolerability of 7D12-s775z in this species. The absence of adverse effects shows that the compound is well tolerated, and the applicability of 7D12-s775z can subsequently be investigated in a dose-escalation study in dogs with spontaneous cancer undergoing tumor resection surgery.

An important point of consideration when performing studies in different species is the cross-reactivity of the targeting agent amongst the different homologues. 7D12 is known to recognize the human variant of EGFR with an affinity in the low nanomolar range (5.4 nM). In the present study, we demonstrated by SPR that this Nb also recognizes recombinant canine EGFR antigen (29.3 nM) and EGFR-mediated binding to canine carcinoma cells was confirmed by flow cytometry. Although the affinity is slightly lower than for the human analogue, mainly because of a faster dissociation rate, such affinity should remain adequate for *in vivo* targeting and imaging purposes. Furthermore, fluorescent labeling of the Nb with s775z did not have a major impact on the functionality of the Nb (K_D_ of 7D12-s775z for human and canine EGFR is 11.1 and 31.6 nM, respectively). To our knowledge this is the first assessment of the cross-reactivity of Nb-7D12 toward canine EGFR and it was the final step before the compound could be released for a preclinical tolerability trial.

Even though dog studies do not require current Good Manufacturing Practices (cGMP) production of the tracer, considerably lowering the costs, the compound was produced under comparable conditions, by cGMP-experienced personnel, using high-grade materials, decontaminated equipment, detailed Standard Operation Procedures, corresponding Work Instructions and systematic Batch Records. Furthermore, the product was thoroughly characterized in terms of chemical purity (SEC, SDS-PAGE, MS, endotoxin-level), identity (MS, Western blot) and spectral characteristics. In the present study, 7D12 was reproducibly labeled with an average DOL of 1, at which the influence of the dye on the functionality and biodistribution of the Nb is kept at a minimum (Baudhuin et al., 2021a). A DOL of 1 could also have been achieved by site-specific conjugation strategies, yet we opted for conjugation of s775z to the Nb via a random strategy on the Nb’s lysines. The rationale hereto is that introduction of genetically encoded tags in the Nb’s structure, e.g., carboxy-terminal cysteine tag for maleimide chemistry, often significantly reduces the production yield of Nbs in expression vectors (Massa et al., 2014). Moreover, enzyme-mediated site-specific conjugation strategies are more challenging to implement in cGMP environments and could result in a more immunogenic product (Agarwal and Bertozzi, 2014). A similar random conjugation strategy was previously used for the clinical translation of chelator-conjugated Nbs to be evaluated as PET tracers for cancer and immune-cell imaging (Keyaerts et al., 2016; Xavier et al., 2019; Gondry et al., 2023). Finally, it was demonstrated that the product was stable for at least 6 months when stored at −20 °C (Supplementary Figures S3). To facilitate distribution for multi-center clinical studies and/or commercialization, a lyophilized kit-formulation might be preferred and could also further prolong shelf-life (Baudhuin et al., 2021b).

The No Observed Adverse Effect Level (NOAEL) of 7D12-s775z was determined to be higher than or at least equal to 19.47 mg/m^2^, our highest tested dose. Doses were calculated based on a modified Fibonacci sequence in which the first dose increases by 100% and the subsequent dose increases by 67% of the preceding one, as described by Le Tourneau et al. (2009). In a future dose-finding study in dogs with EGFR-overexpressing tumors, the doses employed will range from 0.58 to 5.83 mg/m^2^, which equates to a human dosage of 2.5–10 mg per patient. Given that fluorescently labeled Nbs were never investigated as contrast agents in a clinical trial, the choice of dose is based on previous fluorescence imaging studies where similar low molecular weight compounds (e.g., affibodies, peptides, folic acid) were employed on a range from 1 to 18 mg per patient (Samkoe et al., 2017; Tummers et al., 2019; Yamada et al., 2021; Skjøth-Rasmussen et al., 2022; Tanyi et al., 2022). Additionally, the decision for employing a measuring unit of mg/m^2^ was based on the highly variable size difference among canine breeds, in order to achieve more precise dose calculation in this species.

Although potential side effects are minimized due to the single-use nature of the imaging agent, a potential setback of a new drug is organ toxicity, thus renal and hepatic function are customarily assessed. While renal excretion is the main elimination route of Nbs (Keyaerts et al., 2016; Lwin et al., 2020), no signs of nephrotoxicity were detected in serum biochemistry nor urinalysis in our study. Albeit data under publication from our group shows low liver uptake of 7D12-s775z, liver function and coagulation tests were included in this study; no alterations were noted post-injection. Moreover, the most common side effects associated with anti-EGFR mAb administration are related to on-target toxicity (Busam et al., 2001; Hu et al., 2007). The underlying mechanisms are not completely elucidated, but it is likely associated with Ab binding to epithelial receptors, inducing an inflammatory response due to EGFR inhibition (Lacouture, 2006; Madke et al., 2014). Despite repeated administration of 7D12-s775z in this study, none of the dogs included presented dermatological reactions, such as cutaneous lesions, acneiform eruptions, pustules or paronychia.

Another concern when testing a new biopharmaceutical is hypersensitivity to the compound. In our study, there were no immune-related alterations in blood analysis nor acute or subacute clinical signs of hypersensitivity despite repeated administration of dosages above the intended clinical dose. This corroborates a previous study demonstrating the absence of antigen-specific immune responses in mice treated with the same anti-EGFR Nb (Roovers et al., 2007). This outcome is expected given the Nbs’ low molecular weight, which naturally confers reduced immunogenicity and short serum half-life. Moreover, rapid clearance is regarded as an advantage for image-guided surgery, allowing same-day imaging and increased tumor-to-background ratio (TBR) (Krasniqi et al., 2018; Debie et al., 2019). Hence, Ackaert et al. (2021) showed that breast carcinoma patients who were administered intravenously ^68^Ga-labeled anti-HER2 Nbs did not present significant levels of anti-drug Abs. Although radiolabeled Nbs are employed in micro doses, studies investigating the therapeutic potential of Nbs in higher concentrations also reported on the low immunogenic profile (Scully et al., 2019). Regrettably, an immunogenicity assay was not included in this study and should be performed in further investigations.

Finally, before testing a new imaging agent, it is important to estimate the imaging window in which sufficient TBR could be reached. Studies in mice suggest the optimal imaging time with radiolabeled and fluorescently labeled 7D12 Nbs is 1- to 2- hours post-injection (Gainkam et al., 2008; Oliveira et al., 2012; Van Driel et al., 2014; Declerck et al., 2023). In a study where healthy volunteers were imaged after intravenous administration of pafolacianine, stable signal on the skin was observed for at least 6 h after injection and the authors reported that this information was extremely valuable when determining the intraoperative window in cancer patients (Hoogstins et al., 2016). Thus, we acquired fluorescent images of the oral mucosa, a highly perfused epithelial EGFR-expressing tissue. EGFR is physiologically present in the epithelium and hair follicles (Madke et al., 2014), and immunohistochemistry of canine healthy oral mucosa confirmed EGFR expression (own data). Fluorescence intensity reached its peak between the 1- and 2-h time point, steadily decreasing until the 24-h timepoint, likely due to binding to the oral epithelium. Considering that this is the first study to inject fluorescently labeled Nbs in dogs, we expect to confirm the imaging window in a clinical setting. The *in vitro* serum stability assay demonstrated that the compound remains stable during this time frame.

To conclude, the results indicate that 7D12-s775z is well tolerated in dogs, also after repeated administration; fluorescence was observed in EGFR-expressing healthy tissues, and we anticipate higher signal in EGFR-overexpressing tumors. Successful completion of this study paves the way to conduct a clinical trial in canine patients, which in turn will progress the clinical translation of novel tracers in human cancer patients.

## Data Availability

The original contributions presented in the study are included in the article/Supplementary Material, further inquiries can be directed to the corresponding author.

## Abbreviations

BSA, bovine serum albumine; cGMP, current good manufacturing practices; DMEM, dulbecco’s modified eagle medium; DMSO, dimethylsulfoxide; DOL, degree of labeling; EGFR, epidermal growth factor receptor; FBS, fetal bovine serum; FDA, food and drug administration; HRP, horseradish peroxidase; kDa, kilodalton; K_D_, dissociation constant; LAL, limulus amebocyte lysate; mAb, monoclonal antibody; MEM, minimum essential medium; MFI, mean fluorescence intensity; MS, mass spectrometry; Nb, nanobody; NHS, N-Hydroxysuccinimide; NIR, near-infrared; NOAEL, no observed adverse effect level; PES, polyethersulfone; PBS, phosphate-buffered saline; ROI, region-of-interest; SDS-PAGE, sodium dodecyl sulfate–polyacrylamide gel electrophoresis; SEC, size-exclusion chromatography; SPR, surface plasmon resonance; TBR, tumor-to-background ratio.
