# Supplementary material for: Safety assessment of fluorescently labeled anti-EGFR Nanobodies in healthy dogs
Source: Front Pharmacol. 2023 Sep 14;14:1266288. doi: 10.3389/fphar.2023.1266288 (PMC10538052; doi:10.3389/fphar.2023.1266288)
Supplement: Supplementary file 1 [file Table1.DOCX]

# **Supplementary data**

# **SDS-PAGE and Western Blot unlabeled Nb 7D12**


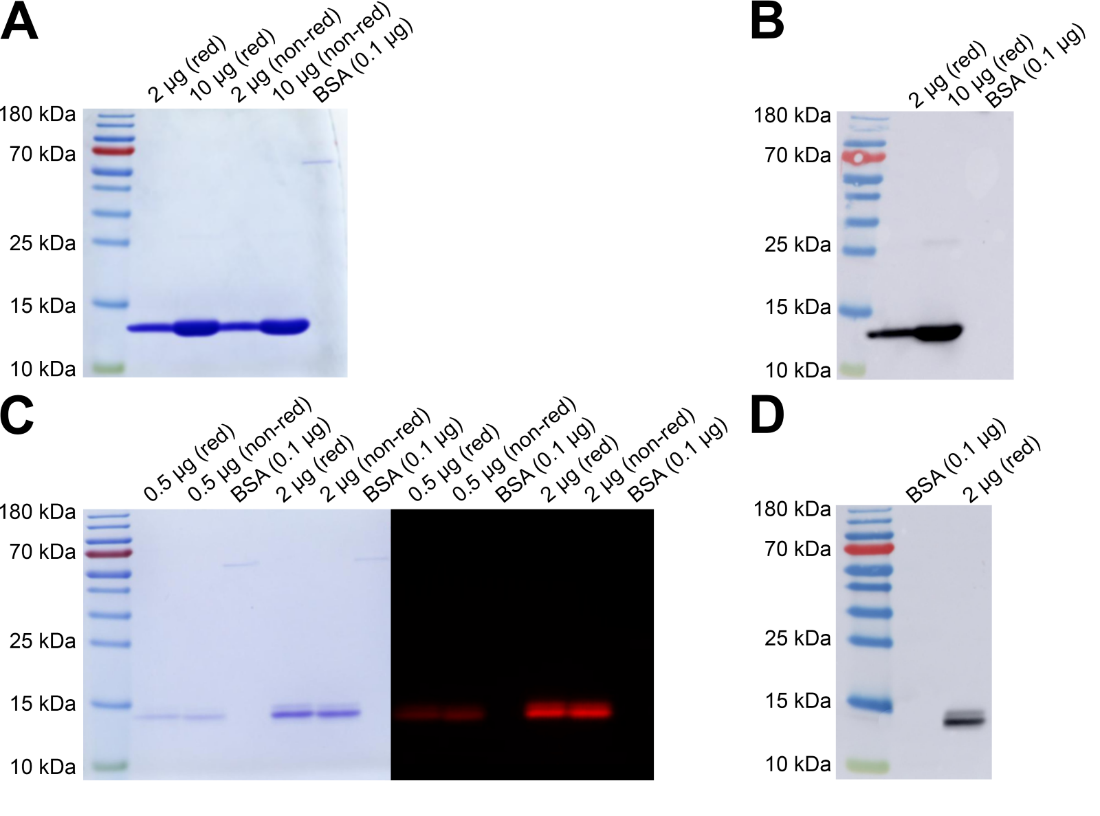


Supplementary figure 1. SDS-PAGE and WB data before and after fluorescent labeling of 7D12. A) Coomassie detection on SDS-PAGE of 7D12 at 2 or 10 µg under reducing (red) or non-reducing (non-red) conditions. Bovine serum albumin (BSA) is included as a standard protein. B) Chemiluminescence Western Blot detection of His-tag on the Nb with Anti-His-tag antibody-HRP of 7D12 at 2 or 10 µg under reducing (red) conditions. C) SDS-PAGE of 7D12-s775z at 0.5 or 2 µg under reducing (red) or non-reducing (non-red) conditions, on the left Coomassie detection and on the right by NIR fluorescence detection. Bovine serum albumin (BSA) is included as a standard protein. D) Chemiluminescence Western Blot detection of His-tag on the Nb with Anti-His-tag antibody-HRP of 7D12-s775z at 2 µg under reducing (red) conditions.

# **Preparative SEC-HPLC of 7D12-s775z**


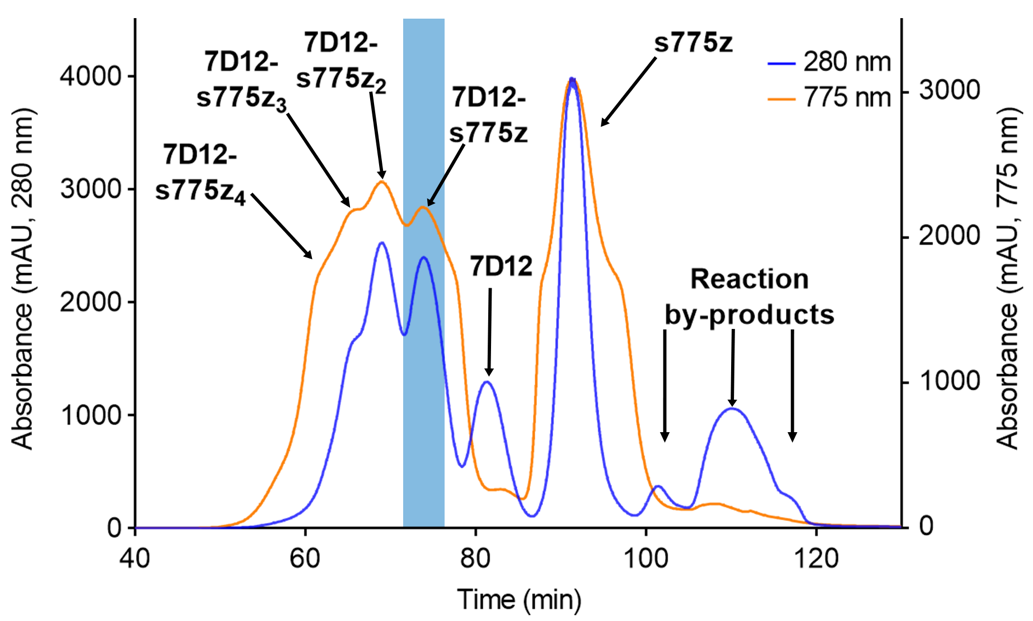


Supplementary figure 2. Chromatogram of preparative-SEC for purification of 7D12-s775z. The readout is in absorption at 280 nm (blue line) and at 775 nm (orange line), the blue bar indicates when fractions containing 7D12-s775z were collected.

# **Stability**


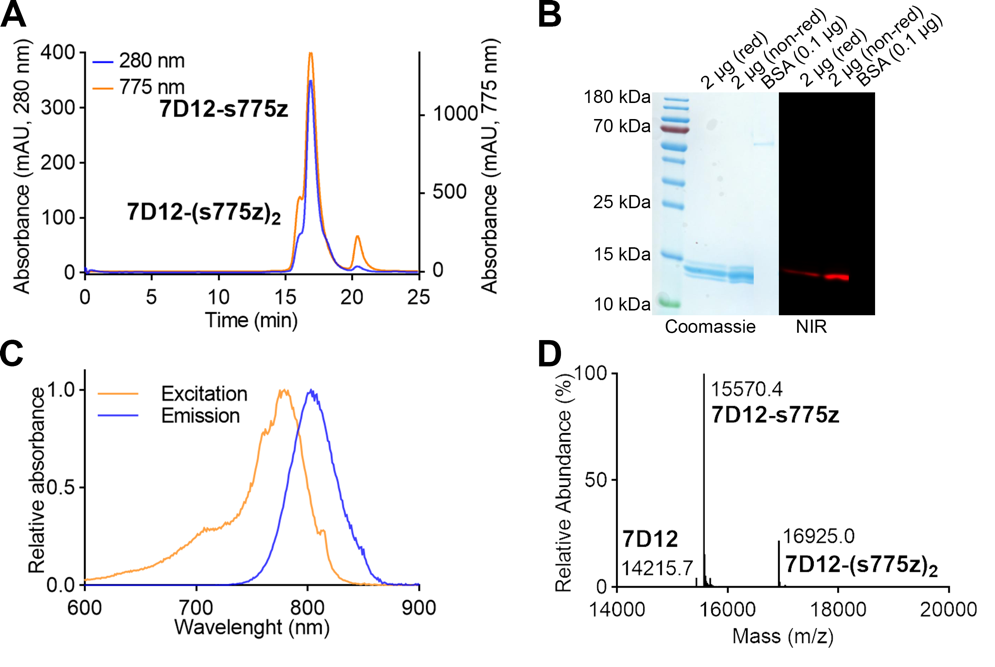


Supplementary figure 3. Stability tests of batch 4 after 6 months of storage at -20˚C. (A) Chromatogram of analytical SEC of 7D12-s775z (200 µg). The readout is in absorption at 280 nm (blue line) and at 775 nm (orange line). (B) Coomassie and NIR-fluorescence detection on SDS-PAGE of 7D12-s775z at 2 µg, under reducing (red) or non-reducing (non-red) conditions. 0.1 µg of bovine serum albumin (BSA) is included as a minimum detection limit. (C) Spectral excitation spectrum (yellow line) and emission spectrum (blue line) of 7D12-s775z (1 µM, PBS). (D) Mass spectrum of 7D12-s775z, mass calculated: 15,587.4, mass found 15,570.4 [M-NH_3_+H]^+^.

# **SPR of unlabeled Nb 7D12 to human and canine recombinant EGFR**


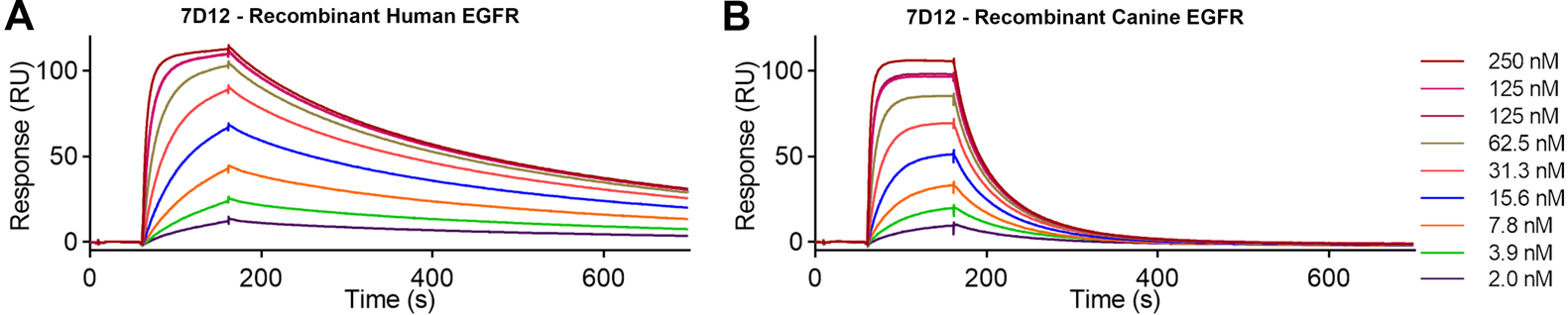


Supplementary figure 4. Assessment of binding kinetics of unlabeled Nb 7D12 to human (A) or canine (B) recombinant EGFR via SPR analysis.

**Flow cytometry analysis of cetuximab binding to human and canine EGFR expressing cancer cells**


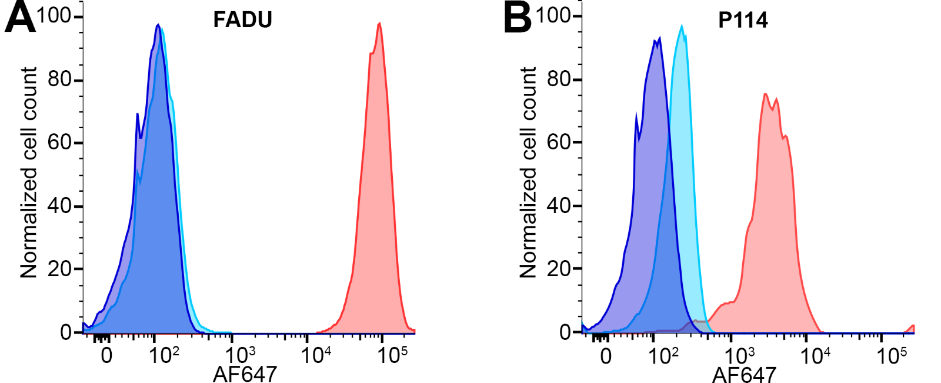


Supplementary figure 5. Flow cytometry analysis on human FADU cells (A) and canine P114 cells (B). Non-stained cells (dark blue), binding of IgG Isotype control-AF647 (light blue) and cetuximab-AF647 (red).

# **QC of 7D12 and 7D12-s775z**

Supplementary table 1. Overview of QC batches.

| Characterization | Specification | 7D12 | 7D12-s775z Batches | | | | | |
| --- | --- | --- | --- | --- | --- | --- | --- | --- |
|  |  |  | **1** | **2** | **3** | **4** | **5** | **6** |
| Yield |  | N/A | 45.1% | 89.4% | 65.2% | 45.6% | 55.1% | 50.7% |
| DOL | [Dye]/[Nb] | N/A | 0.91 | 1.21 | 0.99 | 1.01 | 1.02 | 0.94 |
| Concentration (Nb) | mg/ml | 4.68 | 1.53 | 2.45 | 2.65 | 1.75 | 2.77 | 2.11 |
| Western Blot | Ok/Not ok | Ok | Ok | Ok | Ok | Ok | Ok | Ok |
| SDS Page | Band height (kDa) | 13-14 | 14-15 | 14-15 | 14-15 | 14-15 | 14-15 | 14-15 |
| MS | Main peak | 14215.8 | 15570.4 | 15570.4 | 15570.4 | 15570.4 | 15570.4 | 15570.4 |
| Spectral  characteristics | Max excitation (nm) | N/A | 781 | 780 | 778 | 779 | 781 | 783 |
|  | Max emission (nm) | N/A | 803 | 805 | 805 | 802 | 800 | 803 |
| Endotoxins | EU/mg* | <0.22 | 0.87 | 0.55 | 0.40 | 3.04 | <0.36 | <0.47 |
| SPR  (Human EGFR) | K_D_ (nM) | 5.408 | 8.441 | 13.80 | 13.68 | 8.077 | 10.90 | 11.99 |
|  | k_on_ (10^6^ M^-1^s^-1^) | 0.674 | 0.486 | 0.289 | 0.411 | 0.481 | 0.394 | 0.398 |
|  | k_off_ (s^-1^) | 0.004 | 0.004 | 0.004 | 0.006 | 0.004 | 0.004 | 0.005 |
| SPR  (Dog EGFR) | K_D_ (nM) | 29.34 | 25.72 | 36.54 | 34.53 | 29.06 | 32.50 | 31.14 |
|  | k_on_ (10^6^ M^-1^s^-1^) | 1.091 | 1.032 | 0.745 | 0.811 | 0.980 | 0.899 | 0.974 |
|  | k_off_ (s^-1^) | 0.032 | 0.027 | 0.027 | 0.028 | 0.028 | 0.029 | 0.030 |

*^*^ The endotoxin level was normalized to the concentration of endotoxin units per mg of Nb (EU/mg), and 5 EU/mg was set as the maximally accepted endotoxin level for further use.*

**Serum stability assay**

**
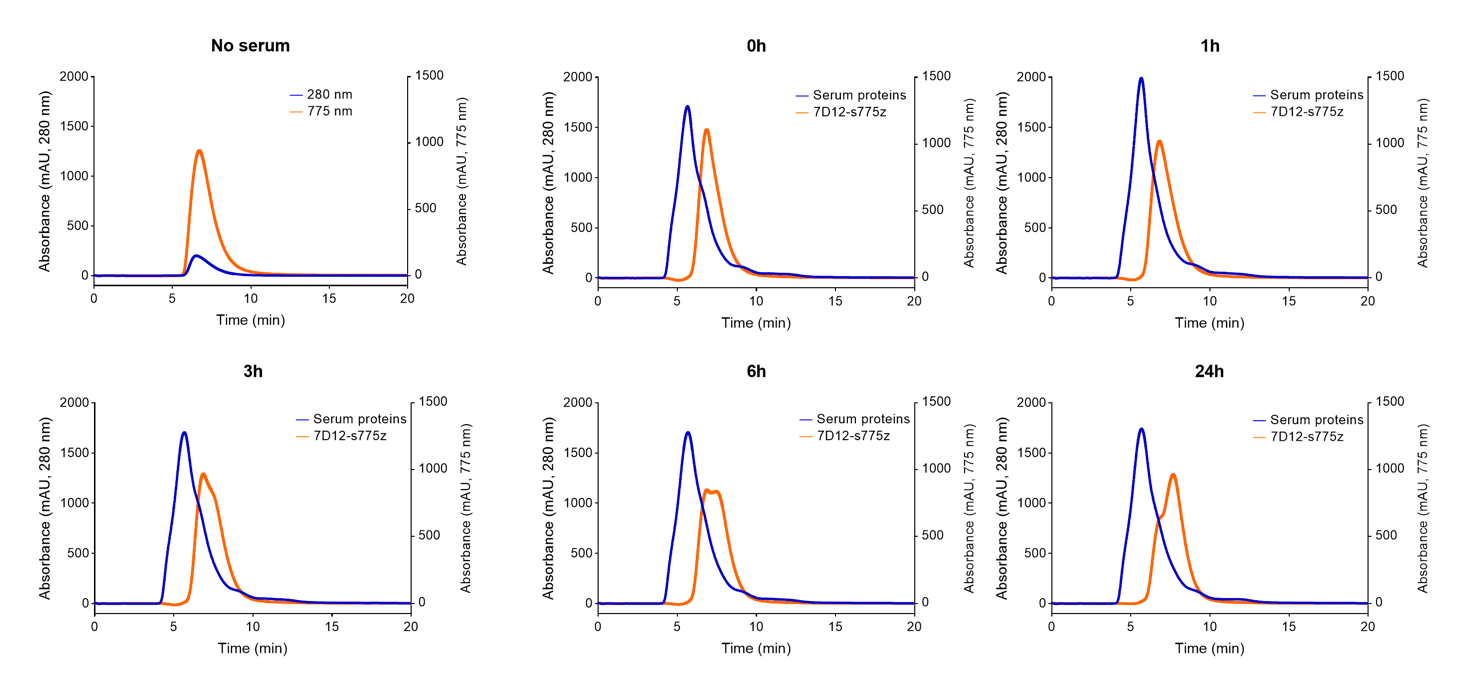
**

Supplementary figure 6. Serum stability assay *in vitro*. Chromatogram of analytical SEC of 7D12-s775z and canine serum. The readout is in absorption at 280 nm (blue line) and at 775 nm (orange line).

**Toxicity assessment**

Supplementary table 2. Overview of the vital parameters following intravenous injections of the first dog. RT: rectal temperature; HR: heart rate; RR: respiratory rate; SBP: systolic blood pressure.

|  | 0 | 2' | 5' | 10' | 30' | 1h | 2h | 4h | 8h | 12h | 18h | 24h |
| --- | --- | --- | --- | --- | --- | --- | --- | --- | --- | --- | --- | --- |
| First dose | | | | | | | | | | | | |
| RT (°C) | 38.1 | 38.1 | 38 | 38 | 37.8 | 37.3 | 37.2 | 36.6 | 37.8 | 37.5 | 38.5 | 37.6 |
| HR (bpm) | 92 | 116 | 92 | 92 | 100 | 96 | 104 | 84 | 88 | 92 | 72 | 104 |
| RR (mpm) | 12 | 12 | 12 | 12 | 16 | 16 | 16 | 16 | 16 | 16 | 16 | 20 |
| SBP (mmHg) | 140 | 110 | 116 | 116 | 132 | 126 | 104 | 128 | 124 | 128 | 120 | 130 |
| Second dose | | | | | | | | | | | | |
| RT (°C) | 37.8 | 37.8 | 38.3 | 38.2 | 37.8 | 37.8 | 37.5 | 37.7 | 37.3 | 38.1 | 37.6 | 38.1 |
| HR (bpm) | 112 | 112 | 116 | 108 | 100 | 116 | 100 | 116 | 100 | 116 | 112 | 120 |
| RR (mpm) | 8 | 8 | 16 | 12 | 12 | 12 | 16 | 16 | 24 | 16 | 12 | 20 |
| SBP (mmHg) | 140 | 140 | 130 | 150 | 140 | 138 | 146 | 148 | 130 | 130 | 130 | 140 |
| Third dose | | | | | | | | | | | | |
| RT (°C) | 37.5 | 37.5 | 37.5 | 37.3 | 37.7 | 37.3 | 36.9 | 37.3 | 37.8 | 37.6 | 37.1 | 37.9 |
| HR (bpm) | 112 | 104 | 104 | 108 | 104 | 108 | 108 | 96 | 96 | 100 | 96 | 96 |
| RR (mpm) | 16 | 12 | 12 | 12 | 16 | 16 | 16 | 12 | 20 | 16 | 12 | 16 |
| SBP (mmHg) | 128 | 126 | 126 | 116 | 122 | 132 | 120 | 128 | 134 | 132 | 128 | 134 |

Supplementary table 3. Overview of the vital parameters following intravenous injections of the second dog. RT: rectal temperature; HR: heart rate; RR: respiratory rate; SBP: systolic blood pressure.

|  | **0** | **2'** | **5'** | **10'** | **30'** | **1h** | **2h** | **4h** | **8h** | **12h** | **18h** | **24h** |
| --- | --- | --- | --- | --- | --- | --- | --- | --- | --- | --- | --- | --- |
| **First dose** | | | | | | | | | | | | |
| RT (°C) | 38.8 | 38.8 | 38.8 | 38.8 | 38.7 | 38.3 | 38.2 | 38 | 37.8 | 38.3 | 37.9 | 38.5 |
| HR (bpm) | 120 | 120 | 120 | 108 | 93 | 96 | 130 | 88 | 116 | 116 | 112 | 96 |
| RR (mpm) | 16 | 16 | 16 | 16 | 20 | 16 | 16 | 20 | 24 | 28 | 24 | 20 |
| SBP (mmHg) | 110 | 110 | 110 | 128 | 134 | 132 | 120 | 128 | 122 | 124 | 122 | 140 |
| **Second dose** | | | | | | | | | | | | |
| RT (°C) | 38.6 | 38.5 | 38.4 | 38.1 | 38.1 | 37.5 | 37.7 | 38 | 38.1 | 37.8 | 37.5 | 38.5 |
| HR (bpm) | 112 | 108 | 100 | 100 | 84 | 84 | 108 | 100 | 120 | 112 | 108 | 124 |
| RR (mpm) | 12 | 16 | 16 | 12 | 16 | 16 | 16 | 16 | 16 | 16 | 16 | 20 |
| SBP (mmHg) | 136 | 140 | 124 | 120 | 130 | 140 | 142 | 138 | 120 | 132 | 124 | 132 |
| **Third dose** | | | | | | | | | | | | |
| RT (°C) | 38 | 38 | 38.1 | 38 | 37.5 | 37.8 | 38 | 37.7 | 37.9 | 37.6 | 37.3 | 37.5 |
| HR (bpm) | 104 | 104 | 96 | 88 | 84 | 96 | 100 | 96 | 104 | 96 | 96 | 100 |
| RR (mpm) | 16 | 16 | 12 | 16 | 12 | 12 | 16 | 12 | 16 | 12 | 12 | 16 |
| SBP (mmHg) | 142 | 142 | 142 | 150 | 126 | 140 | 142 | 140 | 140 | 136 | 130 | 140 |

Supplementary table 4. Overview of the vital parameters following intravenous injections of the third dog. RT: rectal temperature; HR: heart rate; RR: respiratory rate; SBP: systolic blood pressure.

|  | **0** | **2'** | **5'** | **10'** | **30'** | **1h** | **2h** | **4h** | **8h** | **12h** | **18h** | **24h** |
| --- | --- | --- | --- | --- | --- | --- | --- | --- | --- | --- | --- | --- |
| **First dose** | | | | | | | | | | | | |
| RT (°C) | 38 | 38.1 | 38.2 | 38.4 | 38 | 37.8 | 37.7 | 37.3 | 37.6 | 38 | 37.7 | 38 |
| HR (bpm) | 108 | 92 | 120 | 112 | 116 | 120 | 92 | 84 | 104 | 116 | 108 | 140 |
| RR (mpm) | 16 | 16 | 12 | 16 | 16 | 16 | 16 | 12 | 16 | 20 | 12 | 24 |
| SBP (mmHg) | 130 | 128 | 134 | 132 | 134 | 128 | 130 | 120 | 130 | 132 | 128 | 140 |
| **Second dose** | | | | | | | | | | | | |
| RT (°C) | 37.6 | 37.9 | 37.9 | 38.2 | 38 | 37.7 | 37.7 | 37.6 | 37.3 | 37.5 | 37.7 | 37.7 |
| HR (bpm) | 92 | 92 | 92 | 108 | 84 | 92 | 100 | 92 | 96 | 92 | 92 | 100 |
| RR (mpm) | 24 | 16 | 16 | 20 | 16 | 16 | 16 | 20 | 16 | 16 | 12 | 16 |
| SBP (mmHg) | 124 | 128 | 128 | 124 | 140 | 128 | 128 | 138 | 128 | 124 | 128 | 126 |
| **Third dose** | | | | | | | | | | | | |
| RT (°C) | 37.8 | 37.8 | 37.8 | 37.6 | 37 | 37.4 | 37.5 | 37.5 | 37.3 | 37.1 | 37.3 | 37.5 |
| HR (bpm) | 96 | 96 | 80 | 84 | 80 | 80 | 96 | 80 | 96 | 84 | 92 | 96 |
| RR (mpm) | 24 | 24 | 24 | 28 | 24 | 28 | 20 | 24 | 20 | 20 | 20 | 26 |
| SBP (mmHg) | 130 | 130 | 126 | 140 | 140 | 132 | 140 | 138 | 136 | 128 | 130 | 140 |

Supplementary table 5. Overview of the analysis results of the first dog. (I) Baseline values before first injection; (II) 24 hours after first injection; (III) Baseline values before second injection; (IV) 24 hours after second injection; (V) Baseline values before third injection; (VI) 24 hours after first injection

|  | I | II | III | IV | V | VI | Reference |
| --- | --- | --- | --- | --- | --- | --- | --- |
| Hematology | | | | | | | |
| HCT | 48.4 | 49.9 | 47.6 | 45.3 | 43.8 | 47 | 37.3-61.7% |
| RBC | 7.49 | 7.64 | 7.36 | 6.94 | 6.86 | 7.23 | 5.65-8.87 M/μL |
| HGB | 16.8 | 17.4 | 16.7 | 15.9 | 15.6 | 16.4 | 13.1-20.5 g/dL |
| RETIC | 14.2 | 24.4 | 16.2 | 21.5 | 1.2 | 14.5 | 10.0-110.0 K/μL |
| %RETIC | 0.2 | 0.3 | 0.2 | 0.3 | 0.3 | 0.2 | 0-1% |
| MCV | 64.6 | 65.3 | 64.7 | 65.3 | 63.8 | 65 | 61.6 -73.5 fL |
| RDW | 17.3 | 18.7 | 17.6 | 17.6 | 16.6 | 17.2 | 13.6-21.7% |
| MCHC | 34.7 | 34.9 | 35.1 | 35.1 | 35.6 | 34.9 | 32.0 -37.9 g/dL |
| MCH | 22.4 | 22.8 | 22.7 | 22.9 | 22.7 | 22.7 | 21.2-25.9 pg |
| WBC | 5.85 | 6.61 | 6.80 | 7.40 | 5.16 | 6.11 | 5.05-16.76 K/μL |
| LYM | 1.59 | 2.07 | 2.16 | 2.43 | 1.42 | 1.96 | 1.05-5.10 K/μL |
| MONO | 0.34 | 0.6 | 0.42 | 0.58 | 0.27 | 0.47 | 0.16-1.12 K/μL |
| NEU | 3.27 | 3.4 | 3.76 | 3.83 | 3.14 | 3.2 | 2.95-11.64 K/μL |
| EOS | 0.64 | 0.52 | 0.45 | 0.54 | 0.29 | 0.44 | 0.06-1.23 K/μL |
| BASO | 0.01 | 0.02 | 0.01 | 0.02 | 0.04 | 0.04 | 0.00-0.10 K/μL |
| %LYM | 27.2 | 31.3 | 31.8 | 32.8 | 27.5 | 32.1 | 8-21 % |
| %MONO | 5.8 | 9.1 | 6.2 | 7.8 | 5.2 | 7.7 | 2-10 % |
| %NEU | 55.9 | 3.4 | 55.3 | 51.8 | 60.9 | 52.3 | 58-85 % |
| %EOS | 10.9 | 0.52 | 6.6 | 7.3 | 5.6 | 7.2 | 0-9 % |
| %BASO | 0.2 | 0.02 | 0.1 | 0.3 | 0.8 | 0.7 | 0-1 % |
| PLT | 124 | 259 | 243 | 276 | 207 | 243 | 148-484 K/μL |
| Biochemistry | | | | | | | |
| Albumine | 32 | 33 | 31 | 31 | 33 | 33 | 23–40 g/L |
| A/G ratio | 1.1 | 1.1 | 1 | 1 | 1.2 | 1.1 | 0.7-2 |
| ALKP | 67 | 109 | 84 | 66 | 102 | 82 | 23–212 U/L |
| ALT | 48 | 40 | 62 | 66 | 46 | 38 | 10–125 U/L |
| Urea | 2.3 | 6.3 | 4.9 | 7.6 | 3.8 | 6.8 | 2.5–9.6 mmol/L |
| U/C ratio | 9 | 20 | 18 | 26 | 14 | 24 | 10-15 |
| Creatinine | 61 | 77 | 72 | 74 | 70 | 74 | 44–159 µmol/L |
| Globuline | 30 | 29 | 32 | 31 | 28 | 29 | 25–45 g/L |
| Glucose | 4.82 | 5.26 | 5.35 | 5.35 | 5.5 | 5.69 | 4.11–7.94 mmol/L |
| TP | 62 | 62 | 63 | 62 | 61 | 62 | 52–82 g/L |
| Coagulation | | | | | | | |
| PT | 12 | 12 | 14 | 12 | 14 | 14 | 11-17 seconds |
| aPTT | 91 | 93 | 95 | 81 | 82 | 98 | 72-102 seconds |
| Urinalysis | | | | | | | |
| Density | 1.032 | 1.030 | 1.012 | 1.026 | 1.011 | 1.026 | 1.001-1.070 |
| pH | 6 | 7 | 8 | 6.5 | 8 | 7 | 4.5-8.5 |
| WBC | neg | neg | neg | neg | neg | neg | 0-5 |
| Protein | neg | neg | neg | neg | neg | neg | Trace |
| Glucose | neg | neg | neg | neg | neg | neg | Negative |
| Ketone | neg | neg | neg | neg | neg | neg | Negative |
| Urobilinogen | norm | neg | norm | norm | norm | norm | Trace |
| Bilirubine | neg | neg | neg | neg | neg | neg | Trace to 1+ |
| Blood | neg | neg | neg | 250 | 25 | neg | Negative |

.

Supplementary table 6. Overview of the analysis results of the second dog. (I) Baseline values before first injection; (II) 24 hours after first injection; (III) Baseline values before second injection; (IV) 24 hours after second injection; (V) Baseline values before third injection; (VI) 24 hours after first injection.

|  | **I** | **II** | **III** | **IV** | **V** | **VI** | **Reference** |
| --- | --- | --- | --- | --- | --- | --- | --- |
| **Hematology** | | | | | | | |
| HCT | 60.4 | 46.1 | 45.4 | 47 | 48.3 | 45.6 | 37.3-61.7% |
| RBC | 9.23 | 7.09 | 7.02 | 7.19 | 7.48 | 6.99 | 5.65-8.87 M/μL |
| HGB | 20.7 | 16.2 | 16 | 16.4 | 17 | 15.9 | 13.1-20.5 g/dL |
| RETIC | 14.8 | 17.0 | 24.6 | 12.9 | 12.7 | 15.4 | 10.0-110.0 K/μL |
| %RETIC | 0.2 | 0.2 | 0.4 |  | 0.2 | 0.2 | 0-1% |
| MCV | 65.4 | 65 | 64.7 | 65.4 | 64.6 | 65.2 | 61.6 -73.5 fL |
| RDW | 20.1 | 17.7 | 17.4 | 17.9 | 17.7 | 17.2 | 13.6-21.7% |
| MCHC | 22.4 | 22.8 | 35.2 | 34.9 | 35.2 | 34.9 | 32.0 -37.9 g/dL |
| MCH | 34.3 | 35.1 | 22.8 | 22.8 | 22.7 | 22.7 | 21.2-25.9 pg |
| WBC | 5.16 | 6.61 | 5.61 | 5.06 | 4.97 | 4.97 | 5.05-16.76 K/μL |
| LYM | 1.58 | 1.55 | 1.53 | 1.53 | 1.61 | 1.46 | 1.05-5.10 K/μL |
| MONO | 0.25 | 0.48 | 0.34 | 0.45 | 0.38 | 0.41 | 0.16-1.12 K/μL |
| NEU | 271 | 3.57 | 3.47 | 2.90 | 2.82 | 2.92 | 2.95-11.64 K/μL |
| EOS | 0.62 | 0.98 | 0.26 | 0.18 | 0,15 | 0.16 | 0.06-1.23 K/μL |
| BASO | 0 | 0.03 | 0.01 | 0 | 0.01 | 0.02 | 0.00-0.10 K/μL |
| %LYM | 30.6 | 23.4 | 27.3 | 30.2 | 32.4 | 29.4 | 8-21 % |
| %MONO | 4.8 | 7.3 | 6.1 | 8.9 | 7.6 | 8.2 | 2-10 % |
| %NEU | 52.6 | 54 | 61.8 | 57.3 | 56.8 | 58.8 | 58-85 % |
| %EOS | 12 | 14.8 | 4.6 | 3.6 | 3 | 3.2 | 0-9 % |
| %BASO | 0 | 0.5 | 0.2 | 0 | 0.2 | 0.4 | 0-1 % |
| PLT | 162 | 216 | 191 | 189 | 205 | 203 | 148-484 K/μL |
| **Biochemistry** | | | | | | | |
| Albumine | 31 | 28 | 28 | 30 | 30 | 31 | 23–40 g/L |
| A/G ratio | 1.3 | 1.1 | 1 | 1.1 | 1.2 | 1.1 | 0.7-2 |
| ALKP | 81 | 51 | 95 | 85 | 85 | 93 | 23–212 U/L |
| ALT | 34 | 41 | 44 | 50 | 60 | 59 | 10–125 U/L |
| Urea | 3.6 | 5.4 | 3.4 | 4.7 | 3.5 | 5.1 | 2.5–9.6 mmol/L |
| U/C ratio | 14 | 21 | 14 | 16 | 13 | 18 | 10-15 |
| Creatinine | 64 | 65 | 61 | 67 | 67 | 67 | 44–159 µmol/L |
| Globuline | 23 | 25 | 27 | 28 | 26 | 27 | 25–45 g/L |
| Glucose | 4.96 | 5.75 | 5.23 | 5.74 | 5.3 | 5.72 | 4.11–7.94 mmol/L |
| TP | 54 | 53 | 55 | 58 | 56 | 58 | 52–82 g/L |
| **Coagulation** | | | | | | | |
| PT | 16 | 14 | 14 | 17 | 15 | 14 | 11-17 seconds |
| aPTT | 100 | 89 | 90 | 89 | 95 | 100 | 72-102 seconds |
| **Urinalysis** | | | | | | | |
| Density | 1.019 | 1.017 | 1.004 | 1.016 | 1.012 | 1.016 | 1.001-1.070 |
| pH | 7 | 5 | 6.5 | 5 | 6 | 6 | 4.5-8.5 |
| WBC | neg | neg | neg | neg | neg | neg | 0-5 |
| Protein | neg | neg | neg | neg | neg | neg | Trace |
| Glucose | neg | neg | neg | neg | neg | neg | Negative |
| Ketone | neg | neg | neg | neg | neg | neg | Negative |
| Urobilinogen | neg | norm | norm | norm | norm | norm | Trace |
| Bilirubine | neg | neg | neg | neg | neg | neg | Trace to 1+ |
| Blood | 25 | neg | 10 | 10 | 25 | neg | Negative |

Supplementary table 7. Overview of the analysis results of the third dog. (I) Baseline values before first injection; (II) 24 hours after first injection; (III) Baseline values before second injection; (IV) 24 hours after second injection; (V) Baseline values before third injection; (VI) 24 hours after first injection.

|  | I | II | III | IV | V | VI | Reference |
| --- | --- | --- | --- | --- | --- | --- | --- |
| Hematology | | | | | | | |
| HCT | 59.5 | 48.6 | 48.1 | 45.2 | 48.5 | 46.9 | 37.3-61.7% |
| RBC | 8.87 | 7.21 | 7.23 | 6.73 | 7.33 | 6.97 | 5.65-8.87 M/μL |
| HGB | 20.3 | 16.8 | 16.7 | 15.7 | 17.1 | 16.4 | 13.1-20.5 g/dL |
| RETIC | 38.1 | 29.6 | 34 | 19.5 | 35.9 | 24.4 | 10.0-110.0 K/μL |
| %RETIC | 0.4 | 0.4 | 0.5 | 0.3 | 0.5 | 0.4 | 0-1% |
| MCV | 67.1 | 67.4 | 66.5 | 67.2 | 66.2 | 67.3 | 61.6 -73.5 fL |
| RDW | 19.5 | 17.8 | 18.2 | 17 | 17.7 | 16.8 | 13.6-21.7% |
| MCHC | 34.1 | 34.6 | 34.7 | 34.7 | 35.3 | 35 | 32.0 -37.9 g/dL |
| MCH | 22.9 | 23.3 | 23.1 | 23.3 | 23.3 | 23.5 | 21.2-25.9 pg |
| WBC | 8.39 | 9.16 | 7.88 | 6.7 | 7.06 | 7.06 | 5.05-16.76 K/μL |
| LYM | 2.14 | 2.30 | 2.46 | 2.53 | 2.33 | 2.55 | 1.05-5.10 K/μL |
| MONO | 0.38 | 0.66 | 0.49 | 0.47 | 0.43 | 0.57 | 0.16-1.12 K/μL |
| NEU | 3.68 | 4.81 | 3.91 | 2.6 | 3.47 | 3.25 | 2.95-11.64 K/μL |
| EOS | 2.17 | 1.38 | 0.98 | 0.81 | 0.81 | 0.69 | 0.06-1.23 K/μL |
| BASO | 0.02 | 0.01 | 0.04 | 0.03 | 0.02 | 0 | 0.00-0.10 K/μL |
| %LYM | 25.5 | 25.1 | 31.2 | 37.8 | 33 | 36.1 | 8-21 % |
| %MONO | 4.5 | 7.2 | 6.2 | 7 | 6.1 | 8.1 | 2-10 % |
| %NEU | 43.9 | 52.5 | 49.7 | 42.7 | 49.1 | 46 | 58-85 % |
| %EOS | 25.9 | 15.1 | 12.4 | 12.1 | 11.5 | 9.8 | 0-9 % |
| %BASO | 0.2 | 0.1 | 0.5 | 0.4 | 0.3 | 0 | 0-1 % |
| PLT | 177 | 208 | 242 | 220 | 213 | 186 | 148-484 K/μL |
| Biochemistry | | | | | | | |
| Albumine | 31 | 32 | 31 | 30 | 33 | 31 | 23–40 g/L |
| A/G ratio | 1 | 1 | 0.9 | 0.9 | 1.2 | 1 | 0.7-2 |
| ALKP | 69 | 53 | 85 | 79 | 73 | 57 | 23–212 U/L |
| ALT | 47 | 50 | 46 | 46 | 39 | 56 | 10–125 U/L |
| Urea | 2.6 | 6.1 | 2.7 | 6 | 2.5 | 4.7 | 2.5–9.6 mmol/L |
| U/C ratio | 10 | 24 | 11 | 24 | 10 | 19 | 10-15 |
| Creatinine | 60 | 61 | 60 | 63 | 58 | 63 | 44–159 µmol/L |
| Globuline | 32 | 31 | 33 | 32 | 28 | 32 | 25–45 g/L |
| Glucose | 4.8 | 6.46 | 5.76 | 5.88 | 5.51 | 5.32 | 4.11–7.94 mmol/L |
| TP | 63 | 63 | 64 | 62 | 61 | 63 | 52–82 g/L |
| Coagulation | | | | | | | |
| PT | 13 | 14 | 14 | 13 | 14 | 14 | 11-17 seconds |
| aPTT | 93 | 91 | 83 | 91 | 92 | 78 | 72-102 seconds |
| Urinalysis | | | | | | | |
| Density | 1.037 | 1.052 | 1.017 | 1.032 | 1.027 | 1.024 | 1.001-1.070 |
| pH | 6 | 6 | 7 | 6 | 6 | 6 | 4.5-8.5 |
| WBC | neg | neg | neg | neg | neg | neg | 0-5 |
| Protein | neg | neg | neg | neg | neg | neg | Trace |
| Glucose | neg | neg | neg | neg | neg | neg | Negative |
| Ketone | neg | neg | neg | neg | neg | neg | Negative |
| Urobilinogen | 4 | 4 | norm | 4 | 1 | norm | Trace |
| Bilirubine | 3 | 1 | neg | 1 | 1 | neg | Trace to 1+ |
| Blood | 250 | 250 | neg | neg | neg | neg | Negative |
